# The Management of a Patient with a Cervical Disc Herniation: A Case Report

**DOI:** 10.4137/ccrep.s727

**Published:** 2008-05-22

**Authors:** Sherry Tsao, Peter Pidcoe

**Affiliations:** Department of Physical Therapy, Virginia Commonwealth University—Medical College of Virginia, Richmond, VA 23298.

**Keywords:** traction, cervical spine, disc herniation, radiculopathy

## Abstract

**Purpose:**

To present the management of a patient with a cervical disc herniation and illustrate the efficacy of cervical traction as a main form of treatment for cervical disc herniation in conjunction with a home exercise program.

**Background:**

A 71-year-old white female diagnosed with cervical disc herniation at the levels of C5-6 and C6-7 presented to physical therapy with neck pain radiating into the left upper extremity down to the 5th digit of the left hand.

**Treatment:**

The patient reported to outpatient physical therapy for cervical disc herniation and radiculopathy. After initial evaluation she received intermittent cervical traction and was given a home exercise program consisting of cervical lateral flexion stretch, unilateral wall stretch for pectoralis muscles and to continue with her over the door cervical traction.

**Conclusion:**

Cervical traction and a good home exercise program have been shown to reduce cervical disc herniation and its subsequent symptoms.

## Introduction

Intervertebral discs are structures found in between the spinal vertebral bodies from the neck to the sacrum. The discs absorb stress applied to the spine and allow six degrees of freedom where the largest motion present is in the sagittal plane (flexion/extension).^1^ A single disc and its two vertebral bodies does not have much ability to move, however, when put together along the length of the spine, the amount of movement provided is substantial. The discs combined along the cervical spine allow for 50 degrees of flexion, 60 degrees of extension, 45 degrees of lateral flexion, and 80 degrees of rotation.^2^ Each disc is composed of two parts, the nucleus pulposus located in the center and the annulus fibrosis forming the outer portion. The nucleus pulposus provides cushion and it is contained by the annulus fibrosis which also attaches to the vertebral bodies above and below.^3^ The disc provide cushion by being able to take and disperse pressure with compression. In a normal disc the resting intradiscal pressure is between 0.6 and 1.2 MPa. With increased compression on the disc the pressure can rise to 3.5 MPa, which is approximately 250% more than resting pressure.^4^ The amount of pressure and the subsequent movement can be increased and decreased respectively due to the hydration of the intervertebral discs themselves. The ability of the nucleus pulposus to resist compressive loads is due to its high content of proteoglycan aggrecan. However, the structure of the disc does not remain constant throughout life, but changes as we age. The most notable change is the decrease in proteoglycan content of the nucleus pulposus, around 20% in mature adults. This loss is accompanied by a decrease in the water content of the nucleus about 75% in adults, thus leading to the decrease in the ability of the tissue to resist compression.^5^ The loss of ability of the nucleus to distribute the applied loads effectively is thought to play a significant role in the mechanisms of disc degeneration and herniation.^6^

One of the most commonly seen problem in discs are disc herniations, which are abnormal protrusions of a portion of the disc material.^3^ The herniation of an intervertebral disc can have a variety of effects depending on the amount of disc protrusion. If the disc protrudes enough it may impinge on a spinal nerve as it exits the vertebral column; this impingement may cause radiating symptoms into the peripheral extremities depending on the level of the herniation. Cervical disc herniation usually occurs when flexion, extension, rotation, and their combination exceed the strength of the annulus fibrosis and the supporting anterior and posterior ligaments. Cervical flexion has been shown to provide the most compressive force on the disc.^4^ With the combination of these movements, it increases the amount of pressure on the disc thus increasing the chance of a disc herniation.^1^ An effect of decreased hydration in the disc is the decreased strength of the annulus fibrosis. As the discs become less hydrated the annulus fibers become stiffer and less pliable. This can lead to the annulus fibrosis cracking and once compression is applied to the disc the nucleus pulposus could protrude out through the cracked fibers.^6^ In an article by Callaghan et al., it shows that herniations can occur with modest levels of compression and flexion/extension moments but with a high number of motion cycles.^1^ So even with low levels of compression a disc herniation can occur if there is high repetitive activity of the cervical spine. The most common area to have a disc herniation is posterior laterally, this is due to the posterior longitudinal ligaments being relatively weak compared to the other structures containing the disc.^8^ There are seven vertebral bodies in the cervical spine. The most common levels of cervical disc herniation are C5-6 and C6-7, which account for about 90% of all cases.^7^

Cervical traction has been used widely to help relieve neck pain from muscle spasm or nerve compression in rehabilitation setting.^7^ Continuous or intermittent traction has been regarded as an effective treatment for herniated cervical discs because it facilitates widening of the disc spaces. The traction induces pain relief and regression of the herniated discs. Several reports have described the reduction of herniated discs either spontaneously or within the treatment period.^8^ The purpose of this case report is to present the management of a patient with a cervical disc herniation and illustrate the efficacy of cervical traction as a main form of treatment for cervical disc herniation in combination of a good home exercise program for continued management of symptoms resulting from disc herniation.

## Case Description

### Patient history

The patient “ES” was a 71-year-old white female diagnosed with cervical disc herniation at the levels of C5-6 and C6-7. MRI results confirmed this diagnosis. She presented to physical therapy with complaints of left upper extremity tingling and achy feeling with symptoms radiating to the 5th digit of the left hand. The patient reports the upper extremity achy feeling has been present for around three months along with cervical soreness. ES’s past medical history includes a left mastectomy in 1991 which led to adhesive capsulitis in the left shoulder, and a history of neck pain for about 2 years. She reports using an over the door traction unit at home for a total of 15 minutes twice a day with 10 pounds for about a week now. She reports that the home traction has helped maintain her symptoms, where her symptoms have not become worse, yet have not resolved.

### Examination

ES presented to physical therapy with a pain level of 3/10 on the VAS pain scale in her neck, she had an achy feeling in her left upper extremity and some tingling in her 5th digit of the left hand. She reported her pain increases with sleeping and computer work and her pain decreases with traction. The patient presented with tenderness to palpation on the right upper trapezius. All upper extremity dermatomes were equal and symmetrical bilaterally within normal limits. Active ROM of the shoulders was taken in supine (see Table 1). Goniometric measurements were taking for shoulder flexion, abduction, internal rotation, and external rotation. Range of motion measures were performed using standard techniques.^10^

Cervical active ROM was obtained seated (see Table 2). Range of motion measures were performed using standard techniques.^10^ Other cervical ROM was within normal limits.

Upper extremity strength was assessed in a seated position graded on a 0–5 scale and grip strength was obtained using a hand dynamometer (see Table 3).^11^

Special tests assessed included: empty can which was positive on left upper extremity, Hawkins Kennedy test also positive on left upper extremity, cervical compression test with an increase in distal symptoms, and cervical distraction which centralized her symptoms. Lastly ulnar neural tension tested in supine was found to be negative, indicating no adhesion of the ulnar nerve along the path through the upper extremity.

### Evaluation

Evaluation of the results of ES’s initial examination shows decreased ROM of cervical spine and upper extremity, decreased strength in left upper extremity, and pain and tingling in left upper extremity all consistent with cervical disc herniation of C5-6 and C6-7. The results also show a possible impingement at the brachial plexus. It was felt that the patient had a good potential for rehabilitation due to prior level of functional, uncomplicated past medical history, early onset of diagnosis, and good motivation in participating in therapy.

### Intervention

ES received outpatient therapy in a clinic for 7 treatments over a 4 week period. Her intervention began at the end of the initial evaluation where she received intermittent mechanical cervical traction in supine for 15 minutes with cervical flexion of 25 degrees and traction was applied for 45 seconds at 14 pounds and released to 10 pounds for 15 seconds. ES was also given a home exercise program of cervical lateral flexion stretch, unilateral wall stretch for the pectoralis muscles that could be the cause of the impingement at the brachial plexus, and to continue with her home traction unit. Throughout her therapy, ES continued to receive mechanical cervical traction increasing the degree of cervical flexion to 30 degrees, increasing the weight of intermittent traction to 10–16 pounds, and increasing the amount of time to 20 minutes. Chin tucks, standing rows with thera-band, and scapular stabilization exercises were also added in the progression of therapy.

### Outcomes

After 4 weeks of outpatient physical therapy treatment, ES was able to return to her premorbid lifestyle. Her pain level decreased to 0/10 on the VAS scale for the neck and upper extremity. The achy feeling and tingling of the left upper extremity and hand had resolved. ES’s shoulder and cervical active ROM both increased to achieve normal functional limits with no pain. Left upper extremity strength also increased to be comparable to the right upper extremity. Lastly grip strength also increased (see Table 4). ES was able to return to all functional activities that were giving her difficulty prior to treatment such as: sleeping without pain, fastening her bra, performing yard work/gardening, and she was also able to lift using her left upper extremity. The patient was discharged with a home exercise program of all the exercises given during treatment and with instructions to continue cervical traction with her home traction unit.

## Discussion

Many times surgery has been used as a last resort treatment for cervical dysfunction. Conservative treatment is more widely used to treat cervical dysfunctions and is generally believed to alleviate symptoms of cervical radiculopathy. One type of conservative treatment for cervical disc herniation with radiculopathy that has become widely popular in rehabilitation settings, especially involving either muscle spasms or nerve compression has been cervical traction. Cervical traction has been shown to cause widening of the disc spaces allowing for control of patient’s symptoms and also to cause reducibility of disc herniation. In a study by Chung et al., they show how mechanical cervical traction can reduce a patient’s cervical disc herniation. The study included 29 patients that all had cervical disc herniations. Each patient wore a traction device that was constructed of nonmagnetic material that was compatible with the MRI units. The units consist of three main parts: a) a shoulder cover for the base of the device, b) an accordion-shaped middle component that can be expanded by means of air inflation, and c) mandible supports for effective transmission of traction. The anterior portion of the unit was fixed in order for the neck to be placed in a flexed position. The unit applied 30 pounds of traction force. MRI images were taken with the patient’s neck at neutral and then with the device inflated after 10 minutes of application in order to allow time for the traction effect on the herniated disc. The results to this study showed that 21 (72%) patients had complete resolution or partial reduction of the cervical disc herniation with just cervical traction alone. Complete resolution of the herniation was defined as a result in which the disc was completely inside the annulus margin without a residual herniated disc particle. Partial reduction was defined as a more than 50% volume reduction in the herniated disc particle with some residual tissue.^9^ This particular study has shown that cervical traction is a successful conservative treatment for herniated disc. However, it did not have in conjunction a home exercise program, which the author of this paper believes to be an important factor in her patient’s overall rehabilitation. Also no follow-up of the patients in the article was obtained to show how well cervical traction could be in the reduction of disc herniation long term.

Another article by Constantoyannis et al. also shows cervical traction as a successful treatment. The article followed four patients with cervical disc herniations all treated with over the door intermittent cervical traction. The treatment consisted of 45 minutes of traction followed by 15 minutes of no traction with a 5 pound water bag. The treatment lasted 6–8 hours a day for 3 weeks. After the 3 weeks of treatment each patient revealed that they were pain-free with full-range of cervical spine motion. No additional episodes of recurrence were observed over 2 or 3 years depending on the patient.^7^ One limitation to this particular article is that a home exercise program was again not used in the intervention of cervical disc herniation. As the author of this paper has stated before, the use of a home exercise program was also an important factor in her patient’s recovery. However, the study did show the benefits of cervical traction in the long term reduction of cervical disc herniation.

According to the research presented it shows that cervical traction is a successful form of treatment for cervical disc herniations with symptoms radiating into the distal extremities. The author chose to use these types of articles as evidence based treatment for her own patient. Since this article showed that cervical traction was a successful form of treatment for herniated discs and radiculopathy, the author chose cervical traction as the primary form of intervention for ES. The author also chose to use a home exercise program to help in maintaining the symptoms and reducibility of the disc herniation. However, the author was unable to find any research pertaining to the effectiveness of a home exercise program in conjunction to cervical mechanical traction in treatment of a cervical disc herniation.

## Conclusion

This case report shows the importance and usefulness of evidence-based practice for treatment in physical therapy. The conservative treatment of cervical traction has been shown to reduce cervical disc herniation and resolve the radicular symptoms that are involved in other studies along with the case of the author’s patient. However, an important intervention to the resolution of the author’s patient was the combination of a home exercise program in her treatment. The limitation of the case report is that no research was found on the efficacy of a home exercise program in the maintenance of the symptoms and reduction of cervical disc herniation. Another limitation to the case report is that the author is not certain that the resolution of the patient’s symptoms was a combination of cervical traction and the home exercise program or if the patient could have had resolution with just the cervical traction alone like the patients in the studies. Further research can be done to show if the combination of treatment is better or just traction alone.

## Figures and Tables

**Table 1:** Shoulder active ROM initial evaluation.

	Right	Left
**Flexion**	165°	165°
**Abduction**	168°	170°
**Internal rotation**	70°	70°
**External rotation**	70°	65°*

* Increased 5th digit Sx.

**Table 2:** Cervical active ROM initial evaluation.

	Right	Left
**Rotation**	40° with pain	55°
**Lateral flexion**	35°	25° with pain

**Table 3:** Strength of upper extremity and grip initial evaluation.

	Right	Left
**Shoulder flexion**	4+/5	4/5
**Abduction**	4+/5	4/5
**Internal rotation**	5/5	5/5
**External rotation**	5/5	5/5
**Elbow flexion**	5/5	5/5
**Extension**	5/5	5/5
**Finger abduction**	5/5	3+/5
**Grip strength**	22 lbs	16 lbs

3 trials	20 lbs	16 lbs
	18 lbs	16 lbs

**Table 4:** Strength of left hand initial and discharge.

	Initial	Discharge
**Finger abduction**	3+/5	4+/5

**Grip strength**	16 lbs	20 lbs
3 trials	16 lbs	18 lbs
	16 lbs	18 lbs
